# Cost and cost-effectiveness of voluntary medical male circumcision in street-connected youth: findings from an education-based pilot intervention in Eldoret, Kenya

**DOI:** 10.1186/s12981-018-0207-x

**Published:** 2018-11-29

**Authors:** O. Galárraga, P. Shah, M. Wilson-Barthes, D. Ayuku, P. Braitstein

**Affiliations:** 1 International Health Institute, Brown University School of Public Health, Providence, RI USA; 2 Academic Model Providing Access to Healthcare (AMPATH), Eldoret, Kenya; 3 Department of Behavioral Sciences, School of Medicine, College of Health Sciences, Moi University, Eldoret, Kenya; 4 Department of Medicine, School of Medicine, College of Health Sciences, Moi University, Eldoret, Kenya; 5 University of Toronto, Dalla Lana School of Public Health, Toronto, ON Canada; 6 Indiana University, Fairbanks School of Public Health, Indianapolis, IN USA; 7 Regenstrief Institute, Inc, Indianapolis, IN USA

## Abstract

**Background:**

Voluntary medical male circumcision (VMMC) is a critical component of HIV prevention. VMMC policies have achieved initial targets in adult men yet continue to fall short in reaching younger men and adolescents.

**Setting:**

We present the cost and scale-up implications of an education-based, VMMC intervention for adolescent street-connected males, for whom the street has become their home and/or source of livelihood. The intervention was piloted as part of the Engaging Street Youth in HIV Interventions Project in Eldoret, Kenya.

**Methods:**

We used a micro-costing approach to estimate the average cost of a VMMC intervention in 116 street-connected youth. Average cost was estimated per individual and per cohort by dividing total cost per intervention by number of clients accessing the intervention over a 30-day period. Total average costs included direct and support procedure costs, educational costs, and direct research costs. Cost-effectiveness was measured in cost per DALYs averted over a 5 and 10-year period.

**Results:**

The total cost of the intervention was $12,526 over the 30-day period, with an average cost per individual of $108. The direct VMMC procedure cost was approximately $9 per individual. Personnel costs contributed the greatest percentage to the total intervention cost (38.2%), with mentors and social workers representing the highest wage earners. Retreat-related and education costs contributed 51% and 13% respectively to the total average cost, with surgical equipment costs contributing less than 1%. At a cost of $108 per individual, the intervention averted 60166 DALYs in 5 years resulting in a cost per DALY averted of $267.

**Conclusion:**

The VMMC intervention was highly cost-effective in Kenya, despite the additional costs incurred to reach SCY. Further scale-up may be warranted to effectively apply this intervention in comparable populations.

**Electronic supplementary material:**

The online version of this article (10.1186/s12981-018-0207-x) contains supplementary material, which is available to authorized users.

## Background

New HIV infections in Kenya have decreased by more than 19% in adults since 2013 but the number of new infections in young people ages 15–24 has increased by 17% with more than 35,000 annual new infections and 160,000 adolescents currently living with HIV [1–3]. Nearly 29% of all new infections occur among adolescents and AIDS continues to be the leading cause of death among young people in the country [1, 4].

Among young adults in Kenya, certain subpopulations are at an increased risk for HIV infection. Research suggests a ‘hidden hot spot’ of HIV exists among the country’s estimated 300,000 street-connected youth (SCY), defined as any girl or boy for whom the street has become her or his habitual abode and/or sources of livelihood, and who is inadequately protected, supervised or directed by responsible adults [5]. Street-connected youth frequently engage in transactional sex and multiple concurrent partnerships, with rape being an endemic part of street life for girls [6–8]. Alcohol and drug use, low condom use and prior STI infections are largely characteristic of SCY in Kenya and are associated with increased risk for HIV [9]. While these findings are consistent with studies of SCY in other low- and middle-income countries [10–17], research on reaching SCY with effective HIV interventions remains limited, especially in sub-Saharan Africa.

Voluntary medical male circumcision (VMMC) is a critical component of HIV prevention and is widely understood to be cost-effective if provided to males between 15 and 49 years [18–22]. Latest model estimates in several African countries indicate that circumcising youth and adolescents can have a greater cost-savings per HIV infection compared to circumcising adult males alone [23–26]. The strategies Kenya has used to achieve the national target of circumcising 80% of the country’s adult males [27] have routinely overlooked SCY who do not access health services through traditional channels [27, 28]. Programs which employ innovative and locally-tailored approaches to youth and hard-to-reach populations have shown higher circumcision rates, more efficient resource utilization and increased cost-savings [29–32].

This paper presents the costs and scale-up implications of an education-based VMMC intervention in adolescent street-connected males in Eldoret, Kenya. This intervention is part of a larger study to engage street youth in the HIV prevention-care continuum [33, 34].

## Methods

### Study setting

Eldoret is the administrative capital of Uasin Gishu (UG) County with a population of nearly 300,000. Eldoret is home to the Moi Teaching and Referral Hospital (MTRH) and the Academic Model Providing Access to Healthcare (AMPATH) program, a large HIV care and treatment program [35, 36]. In 2010, 51.3% of UG County lived below the national poverty line and approximately 52% of the population were age 20 years or younger [37].

### Sampling and recruitment

Male SCY aged 12–24 who had lived on the street for more than 3 months and spent more than 75% of their days and nights on the street or with other SCY in a shared shelter were approached. SCY who were spending both days and nights on the streets and had limited-to-no guardian contact or who had a caregiver to whom they returned at night were also eligible. Those who had not yet been circumcised were invited to participate in the program. Youth were recruited through a snowball-sampling approach by community leaders and peer navigators who conducted outreach and sensitization activities. Eligible SCY were invited to attend a series of local community assemblies (‘mabaraza’) [38, 39] where the intervention was explained to them in a central location before registering at the start of each intervention cycle. Additional details about the eligible population and recruitment strategy is described elsewhere [33].

### Program description

The VMMC intervention was piloted in three separate cohort groups over a total of 30 days. Each cohort group participated in the intervention over a 10-day period and received the same program components. Forty SCY consented to participate in groups 1 and 2 and 36 SCY consented to participate in group 3. A target of 40 SCY per group was set based on the number of circumcisions that could reasonably be carried out in a 1-day period. Group 1 was conducted in December 2016 and Groups 2 and 3 were conducted in May 2017. The intervention took place on the grounds of a community organization and rehabilitation center for street-connected boys located on the outskirts of Eldoret.

On day 1, SCY consented or assented to the intervention. Social workers conducted baseline quantitative surveys and a clinical officer (CO) and nurse screened participants to ensure suitability for circumcision. Provider-Initiated Testing and Counselling (PITC) staff tested SCY for HIV. On day 2, the CO and nurses conducted the circumcision surgery in accordance with Kenya’s National Voluntary Medical Male Circumcision Strategy [27]. From days 3–5, the SCY recovered from the procedure under the supervision of a nurse and nurse assistant. From days 6–9 trained mentors facilitated educational modules which focused on life skills, sexual health, and HIV prevention. The modules were taught after circumcision because, following local cultural beliefs, males are thought to enter manhood after being circumcised and it was the hope that SCY would be increasingly open to the subjects that were taught during this time. Teaching the modules after the procedure further allowed wounds to heal under medical supervision and in a sanitary setting. At the end of the modules, SCY completed the end-of-intervention quantitative survey and program evaluation. A graduation ceremony was held on day 10 and included community leaders, the County Children’s Officer, and other support staff and relatives. SCY received a t-shirt and a monogrammed bracelet as an award for completing the program. If a participant indicated wanting to leave the streets, a social worker met with him to begin the process of returning the youth to his family home or Charitable Children’s Institution (CCI) [33, 34].

### Costing perspective and procedures

We used a micro-costing approach, from the perspective of the program implementer, to document and account for resources used to implement the VMMC program. Costs were recorded in real time on an ongoing-basis over the course of the intervention. Costs were recorded in Kenyan Shillings (KES) and later converted to United States Dollars (USD) using the average exchange rate during the 30-day intervention period [40]. All costs are presented in USD ($).

All costing information was based on program implementation records; no human subjects’ data were used in this analysis. Costs above the service-delivery level, such as program management, supply chain and staff training costs were excluded from this analysis. These higher-level costs were largely fixed costs for the overall VMMC program, so they are less relevant for a pilot study and micro-costing approach. Experience from other programs suggested that supply chain costs alone could add significantly to total program costs [41, 42].

### Calculations provided in supplemental content

The basic assumptions and program information for this intervention are presented in Table 1. All information and calculations used in this analysis are presented in Additional file 1. All unit costs for resources used (inputs) were based on costs to the program (e.g. program invoices), standard staff and volunteer salary scales, and local retail prices (e.g. minor items procured locally in small amounts). Costs were estimated per individual and per each SCY cohort.

**Table 1:** Basic information for VMMC intervention

	Pilot data	Source
Parameters used in the VMMC intervention		
Costing year		
Group 1	2016, December	
Group 2	2017, May	
Group 3	2017, May	
Eligibility criteria	14–24 years; “street-connected”^a^	
Total SCY recruited	116	
Total VMMCs completed	116	
Personnel salaries (daily rate)^b^		
AMPATH staff		
CO, nurse and social worker	$9.69	
Peer navigators	$4.85	
PITC counsellor	$7.27	
Volunteers		
PITC counsellor, nurse, nurse assistant, mentors, helpers, cooks	$9.69	
Security guard	$14.53	
Direct procedure costs per individual (% total cost)	$8.57 (7.9)	
Procedure support costs per individual (% total cost)	$83.53 (77.3)	
Education cost per individual (% total cost)	$14.07 (13.0)	
Direct research cost per individual (% total cost)	$1.81 (1.6)	
Other information		
Exchange rate		
KES/$ [Dec 1, 2016–May 31, 2017]	103.2	41

	Base value [range]	Source
Parameters used to calculate cost per DALY averted		
Male circumcision prevalence in Kenya, age 10–19 (%)	54.8 [40–100]	1, 44, 46
VMMC direct procedure cost, $ per circumcision	8.57 [8.57–108]	Pilot data; 44, 27, 49
Annual discount rate (%)	3.00 [1–5]	47
HIV incidence—Nyanza County, 2015 (%)	2.0	2
HIV prevalence in males 15–24 years (%)	3.12	2
Adolescent male population in Kenya, 2020 estimates^c^		
Male SCY	149,100	7, KNBS
10–14 years (thousands)	3242.92	KNBS
10–19 years (thousands)	6063.25	KNBS
10–29 years (thousands)	10,473.17	KNBS
DALYs/HIV infection averted	7	20, 43

If no source is listed then values reflect VMMC pilot data

*SCY* street-connected youth, *VMMC* voluntary medical male circumcision

^a^Youth were considered street-connected if (a) they were spending both days and nights on the streets and had limited-to-no guardian contact, (b) were spending a portion or majority of their time on the street and had a caregiver to whom they returned at night, or (c) a combination of these situations at different times

^b^CO: clinical officer; PITC: Provider-Initiated Testing and Counselling

^c^Kenya National Bureau of Statistics; http://kenya.opendataforafrica.org/lpdtibb/kenya-population-by-age-groups

Cost of the intervention was itemized by direct procedure costs, procedure support costs, costs of the education component, and research-related costs. Personnel costs included costs to hire the 24 staff recruited to oversee and implement the intervention. AMPATH staff members were paid a daily volunteer rate according to their existing pay grade and volunteer staff were paid based on responsibilities and the local pay scale.

Materials for meals and firewood for cooking were bought locally and in bulk to keep costs to a minimum. All supplies, consumable and reusable, were obtained through the AMPATH procurement department and purchased from the supplier with the lowest quote.

### Cost-effectiveness implications

To be able to better inform potential scale-up, we estimated the cost per DALY averted for the VMMC intervention using sensitivity analyses. Price estimates, coverage, and years to scale-up were varied and parameters are reported in Table 1.

## Results

Table 1 describes the program implementation parameters and the parameters used to estimate the cost per DALY averted. One hundred and sixteen SCY completed the 10-day program. While it was the goal to recruit only HIV-negative SCY, one participant tested positive for HIV at screening but was allowed to remain in the study to benefit from the education modules and to prevent experiencing stigma amongst his peers. No additional recruitment costs were incurred outside of the personnel costs included in the supplementary materials.

### Direct costs of the VMMC surgical procedure

The total direct cost of the VMMC surgical procedure was $994 for the 116 SCY, $8.57 per individual. The average cost of medical supplies was $648 and contributed the greatest proportion to the direct procedure costs (65.2%). Total personnel costs were $291, contributing 29.2% to the direct procedure cost. One clinical officer, two nurses and two nursing assistants were involved in the clinical procedure. Clinical staff were paid a daily salary of $9.69 and worked six of the 30 intervention days. Equipment costs amounted to 5.6% ($56) of the direct procedure costs.

### Procedure support costs

Procedure support costs contributed more than 75% ($83.53) to the total intervention cost per individual. Support costs included the cost of supplies to prevent infection after the procedure (e.g. underwear, petroleum jelly, shorts), site rental costs, and costs of non-clinical personnel which accounted for 33% of all procedure support costs. The cost of food for the 10-day intervention period was $18.65 per individual, one-fifth of all support costs.

### Costs of HIV education component

Education-related costs included personnel costs for mentors to lead modules on life skills, sexual health, and HIV prevention ($10 per individual), costs of education-related materials, and graduation costs of less than $3 per individual. Education costs contributed 13% to the total intervention cost.

*Research*-*related costs* were negligible ($1.80 per individual) and included personnel costs and printing costs for consent/assent forms.

### Total intervention costs

The total cost of the intervention for 116 SCY over the 30-day period was $12,526, $108 per individual. Personnel costs totaled $4789 for the 30-day period and contributed the largest proportion to the cost of the intervention (38.2%). Non-clinical personnel were the highest wage earners. The cost to employ four education mentors was $1163 and accounted for 9.28% of the total costs. Cooks, general helpers and security guards received the second highest earnings, $827 each for the 30-day period.

After personnel costs, the greatest expenses related to the retreat components. Personal effects totaled $3415 and food amounted to $2163, contributing 27.3% and 17.3% respectively to the total cost. The cost to purchase shorts for the SCY amounted to 11.6% of the total intervention cost, more than the cost of surgical equipment and medical supplies combined.

The cost to conduct 4 days of education modules per intervention group was driven largely by the cost to employ mentors with minimal costs for materials. Research and evaluation costs contributed less than 1.0% to the total program cost.

### Estimated cost-effectiveness

Using the WHO’s definition of cost-effectiveness (that is, the cost per disability-adjusted life-year [DALY] saved is less than three times the per capita gross domestic product) [43], the VMMC intervention was highly cost-effective when targeted to male SCY in Kenya (Table 2). Varying the VMMC intervention cost from $8.57 to $108, the cost per DALY averted ranged from $8.5 to $267 per individual and averted more than 8500 new HIV infections in male SCY within 5 years. Scaling-up the VMMC intervention over 10 years averted 16,000 new HIV infections at a cost per DALY averted of up to $141 per individual and increased the intervention’s cost-effectiveness.

**Table 2:**
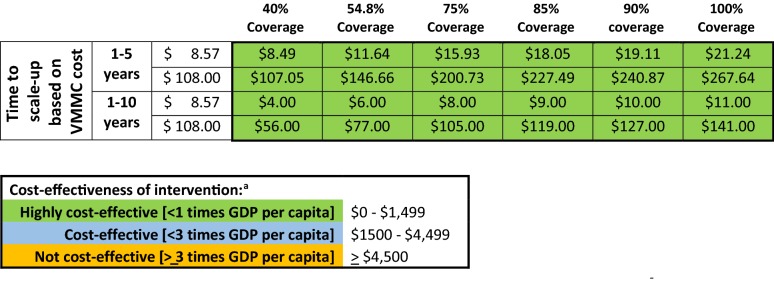
Cost per DALY averted by individual procedure cost and prevalence of circumcision in SCY

^a^Cost-effectiveness ranges are based on the WHO-CHOICE criteria [43]

## Discussion

The direct clinical cost to circumcise high-risk, street connected youth in this program was less than $10 per procedure. This is considerably less than previous estimates for similar VMMC procedures that target high-risk or hard-to-reach populations: $47 per procedure in South Africa [20], $53 in Swaziland [41], and $34–$61 in rural communities in Uganda [44]. We attribute this difference in cost primarily to task-shifting clinical responsibilities to lower-paid staff instead of physicians and to the use of reusable surgical instruments. Based on evidence that task-shifting VMMC procedures reduces costs and maintains comparable levels of quality [45], four healthcare professionals (1 clinical officer and 3 nurses) carried out the procedures such that 2 circumcisions were conducted at the same time which enabled up to 40 circumcisions per day, double the average number of procedures Kenya’s district hospitals can support in a given day [46]. Sterilizing and reusing the instruments between patients limited the costs of new surgical materials and reduced set up time between patients. Additionally, because the circumcisions were carried out during the day in a tent, there were no electricity costs attributed to the procedure.

The major cost drivers of this intervention were related to the retreat structure (to improve outreach and to prevent infection after the procedure) and the education component. The cost to provide three meals a day over the 30-day period was approximately 18% of the total cost, which was significantly more than the cost of surgical equipment and medical supplies. Employing mentors to facilitate the education modules was 10% of the individual intervention cost.

Estimates from the sensitivity analyses indicate that the VMMC intervention is highly cost-effective when administered to street-connected youth, even at a cost of $108 per individual, and these estimates are in line with previous findings from the literature [20, 23, 32]. While differentiated approaches have shown encouraging results for developing VMMC programs that target adolescents [29–31], they often incur greater costs to reach this age group and are limited to the local context [47]. Findings from this pilot intervention are consistent with studies modelling age-specific VMMC programs in Sub-Saharan Africa [23–25] and may support, from a cost-effectiveness perspective, spending more on the differentiated approaches needed to reach these populations.

In terms of strengths, this article contributes a specific example of applying a micro-costing approach to account for the resources used to implement a VMMC intervention for street-connected youth. Since each intervention group remained within the community center for the full 10-day duration, we could more precisely attribute costs to each component of the intervention, in comparison to itemizing costs at a routine clinic where resources are shared across services. The high retention rate allowed cost comparisons between groups and not only at the individual level. Estimates of the cost per DALYs averted indicate that the intervention is highly cost-effective for reaching this high-risk population which may warrant more formal modelling analyses to understand how costs of the intervention will vary in other settings. The main weakness of this study is that we could only observe costs at a single time point due to the cross-sectional nature of the data, and did not attempt to measure causal relationships between the cost of the intervention and HIV outcomes in SCY. Additionally, the costs estimated in this study pertain to a program implemented in the context of a research study. Thus, costs may vary when considering the supply-side costs that would be involved in introducing the intervention in different setting.

## Conclusion

Including outreach and HIV-education activities to engage street connected youth in a VMMC intervention was highly cost-effective in Kenya, despite the additional costs incurred to target this population. Further scale-up may be warranted to effectively apply this intervention in other comparable populations.

## Additional file

**Additional file 1.** ESYH VMMC intervention costs and cost per DALY averted.
